# Three epigenetic information channels and their different roles in evolution

**DOI:** 10.1111/j.1420-9101.2011.02235.x

**Published:** 2011-06

**Authors:** N Shea, I Pen, T Uller

**Affiliations:** * Faculty of Philosophy and Somerville College, University of OxfordOxford, UK; † Theoretical Biology Group, University of GroningenGroningen, The Netherlands; ‡ Edward Grey Institute, Department of Zoology, University of OxfordOxford, UK

**Keywords:** epigenetic inheritance, inherited information, maternal effects, nongenetic inheritance, transgenerational plasticity

## Abstract

There is increasing evidence for epigenetically mediated transgenerational inheritance across taxa. However, the evolutionary implications of such alternative mechanisms of inheritance remain unclear. Herein, we show that epigenetic mechanisms can serve two fundamentally different functions in transgenerational inheritance: (i) selection-based effects, which carry adaptive information in virtue of selection over many generations of reliable transmission; and (ii) detection-based effects, which are a transgenerational form of adaptive phenotypic plasticity. The two functions interact differently with a third form of epigenetic information transmission, namely information about cell state transmitted for somatic cell heredity in multicellular organisms. Selection-based epigenetic information is more likely to conflict with somatic cell inheritance than is detection-based epigenetic information. Consequently, the evolutionary implications of epigenetic mechanisms are different for unicellular and multicellular organisms, which underscores the conceptual and empirical importance of distinguishing between these two different forms of transgenerational epigenetic effect.

## Introduction

Are transgenerational epigenetic mechanisms important for evolution? The evidence is growing that at least they are taxonomically widespread and provide a significant source of phenotypic variation (Jablonka & Raz, 2009). Epigenetic effects can be a cause of evolutionary novelty (Badyaev, 2008; Moczek, 2008; Badyaev & Uller, 2009), they may significantly affect the response to selection (Kirkpatrick & Lande, 1989; Cheverud & Moore, 1994), and could be an adaptation to fluctuating environments (Lachmann & Jablonka, 1996; Rando & Verstrepen, 2007; Uller, 2008). But the evolutionary significance of these mechanisms is still incompletely understood. Does their importance lie in generating novel phenotypes, in mediating transgenerational adaptive plasticity, or in forming an additional inheritance channel in parallel with DNA?

Jablonka & Lamb (1995, 2005) deliberately employ a mechanistic classification scheme in order to highlight the wide variety of nongenetic effects on the phenotypes of future generations. In their usage, all transgenerational epigenetic mechanisms are systems of inheritance. Similarly, Bonduriansky & Day (2009) call all nongenetic transgenerational effects between parents and offspring inheritance. Although this is a legitimate use of the word in its broad sense of ‘things received from a predecessor’, only a subset of epigenetic mechanisms forms a system of long-run inheritance in the way the genome is an inheritance system. Focusing on the type of mechanism involved may obscure such questions about the evolutionary significance of the mechanism (Haig, 2007; Badyaev & Uller, 2009; Shea, 2009; Helanterä & Uller, 2010; Odling-Smee, 2010). This article argues that there are two distinct classes of transgenerational epigenetic mechanisms, which have substantially different evolutionary consequences. Epigenetic effects that would be classified together mechanistically – for example as being based on DNA methylation – will have quite different evolutionary consequences depending upon the way in which they are deployed.

Amongst those transgenerational epigenetic effects that are adaptive and have been selected, we distinguish between what we will call *selection-based* effects and *detection-based* effects. Selection-based effects depend on mechanisms that preserve epigenetic marks reliably down the generations, so that the variant found in the offspring matches that found in the parent (with more or less fidelity). Selection on the phenotypic effects of such epigenetic variants gives rise to adaptations in the same way as selection on genes (Shea, 2007). The second class consists of detection-based adaptive effects. These depend upon mechanisms where the epigenetic variant received by the offspring depends on the environment experienced by the parent. When the resulting phenotype in the offspring is adapted to the environmental feature detected by the parent, these are a transgenerational form of adaptive phenotypic plasticity (commonly referred to as adaptive maternal effects; Uller, 2008). Although the epigenetic mechanisms involved may be identical, the two classes differ in how they generate an adaptive fit between organism and environment. With selection-based effects, the adaptive match between offspring phenotype and environment is due to a history of selection on (more or less) stably transmitted epigenetic variants. With detection-based effects, the match between offspring phenotype and environment is due to the parent having detected an adaptively relevant feature in its environment (Shea, forthcoming a). This information is transmitted to and sets a plastic phenotype in the offspring. (For the plasticity mechanism itself to be an adaptation, there must also have been a history of selection to account for its existence.)

This is only a rough characterisation of the distinction. The purpose of this article is to sharpen the distinction, motivate it by reference to real biological examples, and use it to show how the evolutionary function of epigenetic mechanisms may differ for different organisms and depending on the mechanisms of resetting of epigenetic marks. We start by clarifying the distinction within an abstract formal model. We then show how the distinction has an evolutionary impact in some real biological cases. We put the distinction to work by analysing how it interacts in evolution with a third epigenetic information channel, the one involved in somatic cell inheritance. The role of epigenetic effects in somatic cell inheritance is of immense evolutionary importance as it underpins the emergence of complex multicellular organisms. Other authors have observed that in multicellular organisms, epigenetic marks will only be transmitted between generations provided they do not interfere with somatic differentiation and cell heredity (Jablonka & Lamb, 2005, pp. 148–150). Our contribution is to point out that this conflict may play out differently with selection-based and detection-based effects.

## Epigenetic effects

An epigenetic effect can be defined as an effect of one or several factors on the expression of a phenotype that is heritable but not solely due to changes in DNA (Jablonka & Lamb, 2005; Allis *et al.*, 2007). In this article, we are specifically concerned with a set of mechanisms of epigenetic effects where the unit of transmission is the cell, i.e. cellular epigenetic inheritance (Jablonka & Raz, 2009). Because these effects occur as a result of stable transmission of a particular cellular state, they can occur both within and across generations. Cellular epigenetic inheritance includes different kinds of chromatin marks, such as DNA methylation, histone modification, and several other mechanisms (Jablonka & Lamb, 2005; Jablonka & Raz, 2009). As a mechanism of transmission of cell phenotypes within generations, epigenetic inheritance forms a crucial component of metazoan biology that enables development of different tissues and organs (Goodrich & Tweedie, 2002; Meehan, 2003; Henderson & Jacobsen, 2007; Mohn & Schübeler, 2009).

That cellular inheritance may also pass the bottleneck of meiosis and gamete production is perhaps more surprising, but there is now substantial evidence that epigenetic variants (‘epialleles’) sometimes persist between generations in the absence of DNA variation (although the latter often explains part of the variation in epigenetic marks within populations; Johannes *et al.*, 2008). Epigenetic inheritance in the form of transgenerational stability of DNA methylation has been described in unicellular organisms (e.g. Adam *et al.*, 2008; Csaba 2008), plants (e.g. Molinier *et al.*, 2006; Rangwala *et al.*, 2006; Johannes *et al.*, 2009), and mammals (e.g. Morgan *et al.*, 1999; Rakyan *et al.*, 2003; Crews *et al.*, 2007), although it is unclear to what extent it represents stable transmission of epigenetic variants via germ cells vs. induction *de novo* in each generation through interactions between the maternal phenotype and the environment (reviewed in Rando & Verstrepen, 2007; Youngson & Whitelaw, 2008; Jablonka & Raz, 2009). The extent to which this variation is associated with consistent phenotypic differences under natural conditions, and the fitness consequences thereof, are poorly understood (Bossdorf *et al.*, 2008; Gilbert & Epel, 2009). However, given that differential DNA methylation can have strong effects on morphology, physiology and life history, it seems likely that epialleles can be subject to selection similarly to alleles at genetic loci (Jablonka & Lamb, 2005).

## A formal model of selection-based and detection-based effects

We make the difference between selection-based and detection-based effects more precise by showing how they can be sharply distinguished in a formal model. Our simple model is intended to capture in abstract terms the properties that discriminate between the two channels, rather than to predict the dynamics or equilibria that will be found in any actual populations. We do not model the third epigenetic information channel, involved in somatic cell inheritance, as its properties are already clearly distinguished in the literature.

**Fig. 1:**
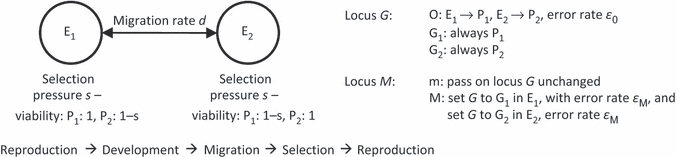
A simple formal model of selection-based and detection-based effects. A population with two phenotypes P1 and P2 is split into two subpopulations living in environments E1 and E2 under selection pressure *s* against nonmatching phenotypes, and migrating between patches at rate *d*.

First, we illustrate the evolution of selection-based effects. Consider a population consisting of two equally large subpopulations in environments E_1_ and E_2_, connected by a low rate of migration *d* (i.e. the probability an individual permanently moves between the two subpopulations before reproducing (Fig. 1)). The life history follows a simple structure with nonoverlapping generations: Reproduction →Development →Migration →Selection →Reproduction. Organisms are haploids and can produce two phenotypes, P_1_ and P_2_. In E_1_, the optimal offspring phenotype is P_1_, in E_2_ it is P_2_. Nonmatching phenotypes have relative fitness 1 − *s*.

Organisms have a locus *G*, which could be considered epigenetic or genetic, with three different possible states (alleles). When in possession of allele O, offspring produce an environment-specific phenotype. They detect their natal environment with error rate ε_O_ (i.e. the probability of incorrectly identifying the natal environment) and develop the matching phenotype accordingly. When in possession of allele G_*i*_ (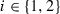), offspring always develop phenotype P_*i*_, regardless of their natal environment. Given a low migration rate *d*, the environment of development is positively correlated with the environment of selection. Furthermore, selection can build up differences in allele frequencies in the two environments because G_1_ is favoured in E_1_ and G_2_ is favoured in E_2_. Thus, there are two sources of information for offspring when dispersal rates are low. If an offspring carries O, it can extract information about the future selective regime from its environment of development. However, G_1_ and G_2_ also carry information as a result of past selection; if an offspring receives G_1_ from its mother it is more likely to be developing in E_1_, and then to be subject to selection in E_1_, than if it receives G_2_ and vice versa (Leimar *et al.*, 2006).

### Selection-based epigenetic effects

We start with a population of organisms of the type O (i.e. without any epigenetic marking) that fix their phenotype by detecting their environment (with error rate ε_O_). When all are of type O, we can calculate the frequencies at equilibrium analytically: the frequency of phenotype P_1_ in environment E_1_ is 1 − (*d*+ *ε*) + 2*dε*. The frequency of P_2_ in E_1_ is therefore (*d*+ *ε*) − 2*dε* (conversely in E_2_– see Supporting Information A7). Thus, for small values of *d* and ε_O_, there will be more of P_1_ than P_2_ in E_1_.

We suppose that G_1_ and G_2_ are epigenetic marks that are perfectly transmitted from parent to offspring and which fix the phenotype of their bearers (to be P_1_ and P_2_, respectively). We ask whether variants G_1_ or G_2_ can invade. We would expect epialleles G_1_ and G_2_ to correlate strongly with environments E_1_ and E_2_ when the selection pressure *s* is high by comparison with the migration rate *d*. If so, offspring may do better by simply adopting the phenotype of their parents (G_1_, G_2_) than they would by detecting the environment for themselves (O), depending on how unreliably they detect the environment for themselves (error rate ε_O_). This intuitive expectation is reflected in our analytic result. The O-equilibrium can be invaded by G_1_ and G_2_ when (ignoring terms that are second order in the small parameter *d*– Supporting Information A8):


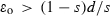
(1)

That is, the G-alleles invade when migration is low and selection is strong in comparison with the error rate with which O-organisms detect their environment. There are no equilibria where the O-allele and G-alleles co-exist, so O goes extinct after invasion of G-alleles and a new equilibrium is reached in which the frequency of G_1_ in E_1_ is 1 − *d*/*s* and of G_2_ in E_1_ is *d*/*s* (and conversely in E_2_– see Supporting Information A10).

We have been assuming that transmission of the G-alleles is perfectly reliable. Although it is not modelled here, lowering the fidelity of transmission (increasing the mutation rate) would act like the migration parameter *d* and would further reduce the correlation between particular alleles and the environment of selection. Low transmission fidelity completely eliminates the opportunity for selection to build up correlations between G-alleles and the selective environment.

The invasion of the O-population by the G-alleles models the selection of an epigenetic effect that is stably transmitted down many generations. Individuals achieve an adaptive match to their environment (P_1_ to E_1_, P_2_ to E_2_) without themselves detecting which environment they are in, and without any individual in their evolutionary history having detected its environment (Shea, forthcoming b). The correlation between epigenetic mark (hence phenotype) and environment is the result of selection, not detection. There is nothing new about this – we noted above that if epialleles are reliably transmitted they may be subject to selection similarly to alleles at genetic loci – but it serves to illustrate our category of *selection-based* effects.

The invasion of O by G is an example of genetic or epigenetic assimilation of a formerly environmentally plastic phenotype (Gilbert & Epel, 2009, p. 375). When the inequality in (eqn 1) is reversed, the G-equilibrium will be invaded by O (see Supporting Information). This exemplifies epigenetic accommodation to stabilize a plastic phenotype (Gilbert & Epel, 2009, p. 384). In fact in our model the variant that produces the well-matched phenotype at the higher frequency at equilibrium is always selectively favoured (Supporting Information A12).

### Detection-based epigenetic effects

Up to this point, our analytic model delivers very similar results to the simulation model of Leimar *et al.* (2006), although this model could also evolve to mixed solutions where individuals rely partly on selection-based information and partly on detecting their selective environment for themselves. Now we extend the model to illustrate the evolution of transgenerational detection-based epigenetic effects (the O-allele was adaptive because of sufficiently accurate detection, but this information was not transmitted between generations). We add a second epigenetic locus *M* that can carry two variants. This locus has an effect on locus *G* (which recall may be considered to be genetic or epigenetic). If *M* carries M the mother assesses her environment with an error 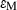 and adjusts the variant on locus *G* so that she sets it to G_1_ in environment E_1_ with probability 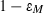 and G_2_ with probability 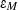, and vice versa for environment E_2_. If she carries variant m she leaves the locus *G* unchanged. In other words, the effect of M is that the mother detects the environment (with error 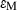) and fixes *G* so that her offspring have a phenotype that matches her environment (and hence the offspring's likely selective environment, provided the migration rate *d* is not too high). The effect of m is that offspring adopt the phenotype that has resulted from long-run selection in their lineage. As before, G_1_/G_2_ is likely to correlate with the local environment when the selection pressure *s* is high and the migration rate *d* is low.

Intuitively, we would expect M to be preferred when the mother can detect her environment reliably (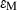 is low) and the information available from selection is poor because the migration rate *d* is high and/or the selection pressure *s* is weak. That is indeed what we find. Variant M invades a population of G-alleles when (to first order in *d*– Supporting Information A13):


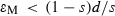
(2)

This is exactly the same condition (replacing 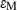 with 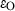) under which the G-equilibrium is invaded by O [the converse of (eqn 1) above]. When *M* takes variant M the mother is doing the same job as the offspring organism did for itself when *G* takes variant O: namely, detecting the environment and setting a plastic phenotype in reliance thereon. The only difference is that with M the information about the identity of the environment is transmitted from mother to offspring. This models cases in which the mother, when mature, can detect which environment she is in more reliably than can the offspring when setting its phenotype at an early stage of development, for example as a germinating seed (Uller, 2008; discussed further below). The invasion of the G-equilibrium by M exemplifies the selection of a detection-based transgenerational epigenetic effect. If G is treated as a genetic locus, then selection of M is an instance of genetic accommodation to an (epigenetically mediated) plastic phenotype.

Thus, this model illustrates how epigenetic mechanisms could be involved in transmitting information between generations in two quite different ways: information generated via natural selection on variants stably transmitted between generations (selection-based effects) and information detected by the parental organism in its environment and transmitted to its offspring (detection-based effects). Both sources of information allow organisms to adapt to local selection and both can be adaptations, but they may have very different consequences for how the organism will evolve.

## Selection-based and detection-based effects in nature

There are very likely to be epigenetically mediated selection-based effects in nature. Experiments show that, at least in unicellular organisms, epigenetic effects are found that are stable for very many, sometimes hundreds, of generations (summarized in Jablonka & Raz, 2009). Natural selection can act on such effects. For example, selection for antibiotic resistance in *Escherichia coli* suggested that rapid evolution of adaptation can occur via epigenetic change (Adam *et al.*, 2008). In the lineage of bacteria grown on an antibiotic medium such as ampicillin, an epigenetic change or changes are selected that cause the bacteria to invest in the metabolic machinery necessary to confer resistance (with its concomitant costs). Offspring receive the adaptive epiallele(s) not because their parent or any ancestor has detected ampicillin in the environment, but because of selection on epigenetic variants favouring a variant that is adapted to ampicillin. Hence, this is a selection-based effect. To the extent that some epigenetic variation at these loci remains in the population, the epialleles will be indistinguishable from genes in a standard heritability analysis (Johannes *et al.*, 2008; Helanterä & Uller, 2010; Tal *et al.*, 2010).

Although they may depend on the same types of epigenetic mechanisms, these cases should not be assimilated with transgenerational phenotypic plasticity in the form of maternal or grandmaternal effects, adaptive cases of which are instead detection-based effects. One of the most convincing examples of an adaptive maternal effect occurs in the herb *Campanulastrum americanum*. This plant can grow as an annual or a biennial and which strategy a seedling adopts depends upon whether its mother grew in woodland understory or in a light gap (Galloway, 2005). Thus, a seedling's life history strategy depends upon some nongenetic maternal effect (although it is not yet clear whether the mechanism is epigenetic in the narrow sense). Experiments manipulating environmental conditions across generations and assessing fitness suggest that this dependence of life history strategy on a maternal effect is an adaptation as the projected population growth is highest when maternal environment and the corresponding timing of offspring germination are matched (Galloway & Etterson, 2007). If so, then evolution has designed a system in which a nongenetic factor is applied that correlates with the maternal environment, and the germinating seedling responds to that correlate by producing an appropriate developmental outcome (annual vs. biennial). That is a detection-based effect. The mother passes epigenetic information to its offspring about their likely environment, and the offspring produce an adaptively appropriate phenotype in response. Other potential candidates for adaptive maternal effects that may involve epigenetic mechanisms include maternal temperature or nutrient effects on offspring development and reproductive strategies in arthropods (e.g. Alekseev & Lampert, 2001), maternally mediated metal tolerance in bryozoans (Marshall, 2008), transgenerational effects of herbivory on seed development and plant morphology (e.g. Agrawal, 2001), and effects of prenatal hormones on offspring morphology, physiology, behaviour and life history in vertebrates (reviewed in e.g. Groothuis *et al.*, 2005).

## Interference between epigenetic information channels

The framework outlined above preserves the insight that selection-based and detection-based effects are solutions to a common informational problem – the problem of producing a phenotype which adaptively matches the organism's environment. It also makes an important distinction based on how this information was generated. In this section, we examine some ways in which those informational roles may conflict with epigenetic mechanisms’ role in multicellular organisms of carrying information for the purpose of somatic cell inheritance.

In unicellular organisms, there is no somatic cell inheritance so there is no conflict. The way is clear for epigenetic mechanisms to form the basis of a second inheritance system alongside DNA (Jablonka & Lamb, 1995, 2005; Helanterä & Uller, 2010), although with important differences in mutation rates and so in the time course of selection. However, in multicellular organisms epigenetic mechanisms are crucial for somatic differentiation and cell heredity (Goodrich & Tweedie, 2002; Meehan, 2003; Henderson & Jacobsen, 2007; Mohn & Schübeler, 2009). Epigenetic marks are passed on when the cell divides, giving descendant cells the same identity. In this way, the epigenetic marks involved in cell heredity carry adaptively relevant information down through many generations of somatic cells.

The ancestral form of multicellularity did not have clearly differentiated germline cells. Embryogenesis proceeded from somatic cells (Buss, 1987). Whatever epigenetic mechanisms are responsible for making different somatic cells have the particular identity they do, those mechanisms have to be reset to a pluripotent state when those cells form the embryo of an offspring organism – otherwise a new plant developing from a leaf cell would consist only of leaf cells. It follows that the selection-based information that has been transmitted through very many generations of ancestors must also be preserved during individual development in the somatic cells that eventually undergo embryogenesis. Such cells must therefore carry two types of information: about what cell type to become in the plant (information about cell heredity) and about how to make a whole new plant in embryogenesis (selection-based information). A single epigenetic locus cannot carry both sorts of information at the same time.

This means that somatic embryogenesis only works if selection-based information has not been discarded. In several species with early segregation of the germline, including some nematodes, unneeded coding DNA is indeed eliminated in various somatic cell lines (Müller *et al.*, 1996; Müller and Tobler, 2000). This is obviously incompatible with somatic embryogenesis. In our framework, it would interfere with a channel of selection-based information – a channel by which information, produced by selection over generations of ancestors, is reliably preserved during the lifetime of the organism and transmitted to the next generation. Epigenetic marking provides a solution to this problem as it does not alter the underlying DNA sequence. Even differentiated somatic cells contain all the genetic information that has been built up through a history of selection. The epigenetic marks involved in cell differentiation and heredity can be reset when a somatic cell founds a new organism. The question is whether this process is compatible with epigenetic marks forming the basis of selection-based and detection-based effects?

The answer depends upon how resetting is achieved. If the epigenetic marks used in somatic cell inheritance are reset piecemeal, then epigenetic loci that are not involved in somatic cell differentiation could be the basis of selection-based effects. As Jablonka & Lamb (2005, p. 149), have argued a fertilized egg could have epigenetic marks that come into play only during leaf-cell development, leading to a new variety of leaf cells. If such marks were reliably transmitted down the generations, then the new variant could be selected, provided it did not interfere with other aspects of somatic cell differentiation and development (Jablonka & Lamb, 2005, pp. 149–150) – a constraint that applies equally to genes. There would be no conflict between information channels: some epigenetic loci would be used to transmit information about cell heredity between lineages of cells in the soma of a single organism; other epigenetic loci would be used to transmit selection-based information down lineages of organisms.

**Fig. 2:**
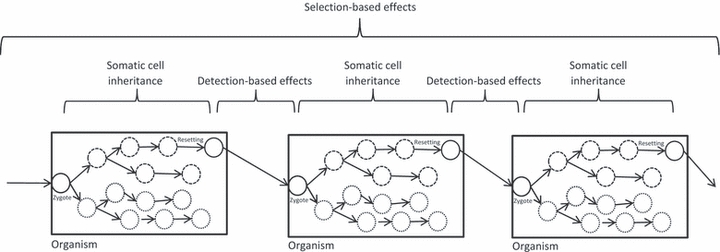
Somatic cell inheritance takes place between cells in the lifetime of an organism. Detection-based effects are based on epigenetic factors which are sensitive to the parent's environment and are transmitted from parent to offspring. Selection-based effects are generated by selection over and are transmitted down many generations of organisms, subsuming the timescale of both detection-based effects and somatic cell inheritance.

The potential conflict arises if resetting does not occur piecemeal. If all epigenetic marks of a particular sort – all methylation marks, say – are reset during somatic embryogenesis, then there is no prospect of any of those marks forming the basis of selection-based effects. Global resetting would have the effect of treating methylation marks as a dedicated channel for somatic cell inheritance, blocking its use for selection-based effects. From one point of view, this observation is rather pedestrian: of course epigenetic marks cannot be the basis of long-run selection if they are reset in each generation. It is more interesting from the evolutionary point of view because it emphasizes a trade-off between the benefits of multicellularity using a given epigenetic mechanism for cell differentiation and heredity, and the benefits of using those same mechanisms as a channel for selection-based effects. This evolutionary trade-off represents a conflict between information channels (see Fig. 2).

Now consider detection-based epigenetic effects. These effects too will be adaptive only if they do not interfere with somatic development. In contrast to selection-based effects, however, there is no obvious conflict between detection-based effects and global resetting of epigenetic marks. Detection-based effects require a channel that can carry information between generations. Somatic cell inheritance requires a channel that can carry information down lineages of cells within a generation. Those channels are not automatically in conflict because they operate at different times (see Fig. 2). Detection-based effects require a mechanism for applying a particular epigenetic mark for the purpose of transgenerational phenotypic plasticity. If there is global resetting of epigenetic marks in the zygote or later in development (reviewed in Feng *et al.*, 2010), maternally mediated alterations to those marks can occur after global resetting. For example, the prolonged physical relationship between mother and offspring that is typical of mammals provides substantial scope for (potentially adaptive) maternal induction of epigenetic changes in somatic cells, which may result in transgenerational persistence of an environmentally induced effect (e.g. Weaver *et al.*, 2004; see also Badyaev & Uller, 2009). Thus, detection-based effects may involve the very same mechanism that is used for somatic cell inheritance. And if epigenetic marks are reset piecemeal, then a locus may be used to induce a detection-based effect expressed in early development in offspring, before being recruited to play a role in cell differentiation and heredity later in the life of the organism.

In summary, if resetting always occurs piecemeal, then detection-based and selection-based effects are equally compatible with somatic embryogenesis, subject to the generic constraint that they must provide a net advantage at the level of the whole organism and the specific constraint that a particular locus cannot be used to transmit both information for somatic cell heredity and selection-based information at the same time. However, in organisms where there is global resetting of a whole category of epigenetic marks, as seems to be the case in mammals (Sasaki & Matsui, 2008; Feng *et al.*, 2010; but see Borgel *et al.*, 2010), that will stop those mechanisms being the basis of selection-based effects, but does not in principle prevent them being a mechanism for transmitting detection-based effects.

## Empirical patterns and alternative explanations

The data on transgenerational epigenetic inheritance summarized in Jablonka & Raz (2009) suggest that epigenetic effects are common and taxonomically widespread. Although data on the taxonomic distribution of epigenetic inheritance have not been collected in a systematic way, stable transgenerational inheritance of epialleles over tens or hundreds of generations has been observed in unicellular organisms (e.g. Csaba *et al.*, 1982; see online table in Jablonka & Raz, 2009). In multicellular organisms, transgenerational transfer of epigenetic variants seems to be most common in plants and fungi (Jablonka & Raz, 2009). Typically, these variants are only stable for a limited number of generations, although studies that have investigated long-term stability are rare. For example, experimentally induced DNA methylation in *Arabidopsis thalina* reverts to the wild-type epigenetic state within two to five generations at half the methylatable sites (Johannes *et al.*, 2009; Reinders *et al*., 2009; Teixeira *et al.*, 2009). At the other half, DNA methylation is transmitted for at least eight generations (being the length of the experiment rather than an observed limitation).

Overall, these data are consistent with our prediction that stable long-term inheritance of epialleles could form the basis of selection-based effects in unicellular organisms. It also supports the idea that, in multicellular organisms, there is a potential conflict in using the same epigenetic mechanism both for somatic cell inheritance and for carrying selection-based information. This conflict is more acute if epigenetic marks are globally reset in each generation, which is indeed suggested by the extensive reprogramming of epigenetic marks observed in animal germ cells post-fertilization (Sasaki & Matsui, 2008; Feng *et al.*, 2010; Popp *et al.*, 2010). The shorter term detection-based effects, occurring over a single or multiple generations (Jablonka *et al.*, 1995; Jablonka & Lamb, 1995), may be more common in multicellular organisms, as there is less conflict between somatic cell inheritance and detection-based epigenetically mediated transgenerational adaptive plasticity.

The existing literature contains two further explanations for the distribution of epigenetic mechanisms, which are complementary to the considerations about conflicts between information channels considered here (Jablonka & Lamb, 1995, 2005; Jablonka & Raz, 2009). There is a developmental argument and a selective argument, both to the effect that epigenetic effects will be less common in animals than in plants and fungi.

The developmental argument is that, amongst the multicellular organisms, there is more scope for passing on epigenetic effects to offspring in plants and fungi, where there is late separation of germ line and soma, than there is in those animals where segregation between germ line and soma occurs early (like mammals). The same logic would suggest that there is also more scope for passing on epigenetic effects in unicellular organisms than there is in animals with preformation of the germ line. In our view, this developmental argument applies differently to detection-based and selection-based effects. We argued above that, given somatic embryogenesis, the same epigenetic locus cannot carry both selection-based information between generations and information about somatic cell identity within a generation. Moreover, with late separation of the germ line, the kinds of epigenetic modifications transmitted are more likely to be environmentally induced, rather than faithfully replicated down the generations. This is again more suited to detection-based effects than to selection-based effects. This suggests one reason why in plants (with somatic embryogenesis), selection-based effects may be less common than detection-based effects (i.e. adaptive effects at short to medium timescales, relative to the timescale of cycles of environmental change; e.g. Molinier *et al.*, 2006; Verhoeven *et al.*, 2010).

The second consideration is a selective argument. Animals may have less need for epigenetic inheritance because they have a nervous system and mobility to allow them to adapt to local conditions (Jablonka & Lamb, 1995, 2005; Jablonka & Raz, 2009). In a cyclic environment that is stable for several generations and then changes state, plants may have particularly good reasons for relying on epigenetically based detection-based information to produce an adaptive phenotype that lasts for several generations (medium-term detection-based effects). Furthermore, animal mobility, and the learning which is mediated by the nervous system may reduce the predictability of the environment encountered by offspring, which makes information detected by parental organisms less reliable when transmitted to offspring.

We agree that these considerations may count against detection-based epigenetic effects being as important in animals as they are in plants. However, they do not tell us whether stable, long-term epigenetic transmission, expressed as selection-based effects, should be common or rare in animals. The limited evidence so far suggests that global resetting of epigenetic variants is more extensive in animals than in plants and that there are few epigenetic marks that are reliably transmitted over the long term in animals (Feng *et al.*, 2010) – so selection-based effects will be rare. The argument about nervous systems and mobility does not tell us why that should be so but the framework outlined here gives us some insight. There is a conflict between using epigenetic mechanisms for somatic cell inheritance and using them as the basis for reliable transmission of phenotypic variants on which selection can act. In many animals at least, evolution appears to have resolved that conflict in favour of the benefits of specialization and multicellularity, with associated global resetting of epigenetic marks in germ cells and during embryogenesis. In other taxa where epigenetic marks can accumulate selection-based information (e.g. unicellular organisms), this informational conflict is resolved in favour of epigenetic mechanisms forming another system of long-run inheritance alongside genes.

The results summarized by Jablonka & Raz (2009) are really too partial and selective at the current stage of enquiry to license any conclusions about the distribution of epigenetic inheritance across taxa. Even given partial data, however, the discussion above should illustrate the importance of distinguishing between selection-based and detection-based epigenetic effects for a proper understanding of the empirical patterns observed.

## Conclusion

Epigenetic mechanisms are fundamental to somatic cell inheritance. There is increasing evidence that they also are important for information transfer across generations. We argue that an important distinction can be made between selection-based effects, which carry adaptive information in virtue of selection over many generations of reliable transmission, and detection-based effects, which carry information about an environmental feature detected by a parent. Both effects allow offspring to produce an adaptive phenotype, but the way in which information is generated differs.

In multicellular organisms, a conflict arises with the use of epigenetic mechanisms to transmit information about cell identity in somatic cell inheritance. In organisms with somatic embryogenesis, selection-based information must be preserved in somatic cells if they are to be capable of generating a whole differentiated multicellular organism in the next generation. Loci carrying such selection-based information cannot simultaneously carry the information needed for somatic cell inheritance. Furthermore, if a particular epigenetic mechanism is globally reset during the lifetime of the organism, then no selection-based information could accumulate using that mechanism. There are no such conflicts between somatic cell heredity and detection-based information. Accordingly, where there are transgenerational epigenetic effects in multicellular organisms, we would expect to find short- to medium-term detection-based effects to be more prevalent than long-term selection-based effects akin to those transmitted on the basis of DNA.

## Supporting information

**Data S1** Further model details.

As a service to our authors and readers, this journal provides supporting information supplied by the authors. Such materials are peer-reviewed and may be re-organized for online delivery, but are not copy-edited or typeset. Technical support issues arising from supporting information (other than missing files) should be addressed to the authors.
