# Supplementary material for: Three epigenetic information channels and their different roles in evolution
Source: J Evol Biol. 2011 Jun;24(6):1178–87. doi: 10.1111/j.1420-9101.2011.02235.x (PMC3116147; doi:10.1111/j.1420-9101.2011.02235.x)
Supplement: Supplementary file 1 [file jeb0024-1178-SD1.pdf]

Construction of the model

The parameters of the model are described in main text and illustrated in Figure 1. The aim of the model is to formalize the ways by which epigenetic mechanisms can carry information and thereby contribute to the evolution of adaptation; it is not intended as a detailed model of the evolution of plasticity for which we refer to existing theory (summarized in Berrigan & Scheiner 2004).

With two environments, two phenotypes, three alleles at the *G* locus and two alleles at the *M* locus there are $|\{E_1,E_2\}|\times|\{O,G_1,G_2\}|\times|\{m,M\}|\times|\{P_1,P_2\}|=24$ possible individual states. Since a G_1-carrier never develops P_2, and vice versa, there are only 16 individual states that may occur with positive frequency. The model keeps track of the 16 frequencies, censused just after migration and before viability selection. The life cycle is therefore tracked in the following order:

Selection → Reproduction and Marking → Development → Migration

The state frequencies are stored in the vector $\boldsymbol{p}=(p_1,\ldots,p_{16})$ according to the table

G	E	P	$M=$ m	$M=$ M	Viability
O	E_1	P_1	p_1	p_9	1
O	E_1	P_2	p_2	p_{10}	$1-s$
O	E_2	P_1	p_3	p_{11}	$1-s$
O	E_2	P_2	p_4	p_{12}	1
G_1	E_1	P_1	p_5	p_{13}	1
G_1	E_2	P_1	p_6	p_{14}	$1-s$
G_2	E_1	P_2	p_7	p_{15}	$1-s$
G_2	E_2	P_2	p_8	p_{16}	1

During each of the four phases of the life cycle the state frequency changes can be described by a specific 16×16 matrix. Viability selection can be described by a diagonal matrix $\boldsymbol{S}$, with diagonal specified according to the last column of the table.

Marking changes state frequencies according to multiplication by the block matrix

$$\boldsymbol{M}=\begin{bmatrix}\boldsymbol{I}_8 & & \\ & \boldsymbol{O}_4 & \boldsymbol{O}_4 \\ & \boldsymbol{M}_4 & \boldsymbol{M}_4\end{bmatrix}. \quad \text{(A1)}$$

The 8×8 identity matrix $\boldsymbol{I}_8$ corresponds to the absence of marking by *m*-mothers. The 4×4 zero-filled matrix $\boldsymbol{O}_4$ says that *M*-mothers have no offspring with the O-allele at the *G*-locus, while the 4×4 matrix $\boldsymbol{M}_4$ encodes differential marking according to the rule

$$\mathrm{ME}_i G \mathrm{P}_i \rightarrow \begin{cases} \mathrm{ME}_i \mathrm{G}_i \mathrm{P}_i & \text{with probability } 1-\varepsilon_\mathrm{M} \\ \mathrm{ME}_i \mathrm{G}_{\{1,2\}\backslash i} \mathrm{P}_i & \text{with probability } \varepsilon_\mathrm{M} \end{cases} \tag{A2}$$

which translates into

$$\boldsymbol{M}_4 = \begin{bmatrix} 1-\varepsilon_\mathrm{M} & 0 & 1-\varepsilon_\mathrm{M} & 0 \\ 0 & \varepsilon_\mathrm{M} & 0 & \varepsilon_\mathrm{M} \\ \varepsilon_\mathrm{M} & 0 & \varepsilon_\mathrm{M} & 0 \\ 0 & 1-\varepsilon_\mathrm{M} & 0 & 1-\varepsilon_\mathrm{M} \end{bmatrix}. \tag{A3}$$

Phenotypic development changes the state frequencies according to the matrix $\boldsymbol{D} = \mathrm{diag}(\boldsymbol{D}_4, \boldsymbol{I}_4, \boldsymbol{D}_4, \boldsymbol{I}_4)$, where

$$\boldsymbol{D}_4 = \begin{bmatrix} 1-\varepsilon_\mathrm{O} & 1-\varepsilon_\mathrm{O} & 0 & 0 \\ \varepsilon_\mathrm{O} & \varepsilon_\mathrm{O} & 0 & 0 \\ 0 & 0 & \varepsilon_\mathrm{O} & \varepsilon_\mathrm{O} \\ 0 & 0 & 1-\varepsilon_\mathrm{O} & \varepsilon_\mathrm{O} \end{bmatrix}. \tag{A4}$$

Finally, migration affects state frequencies according to the matrix $\boldsymbol{B} = \mathrm{diag}(\boldsymbol{B}_\mathrm{O}, \boldsymbol{B}_\mathrm{G}, \boldsymbol{B}_\mathrm{O}, \boldsymbol{B}_\mathrm{G})$, with

$$\boldsymbol{B}_\mathrm{O} = \begin{bmatrix} 1-d & 0 & d & 0 \\ 0 & 1-d & 0 & d \\ d & 0 & 1-d & 0 \\ 0 & d & 0 & 1-d \end{bmatrix}, \boldsymbol{B}_\mathrm{G} = \begin{bmatrix} 1-d & d & 0 & 0 \\ d & 1-d & 0 & 0 \\ 0 & 0 & 1-d & d \\ 0 & 0 & d & 1-d \end{bmatrix}. \tag{A5}$$

Thus, non-normalized state frequency changes from the beginning to the end of a life cycle are described by the matrix product $\boldsymbol{A} = \boldsymbol{BDMS}$ and the normalized changes from one generation to the next are given by

$$p_i' \equiv F_i(\boldsymbol{p}) = \frac{(\boldsymbol{Ap})_i}{\sum c_j p_j}, \quad i = 1 \ldots 16 \tag{A6}$$

where $c_j = \sum_i A_{ij}$ is the jth column sum of $\boldsymbol{A}$.

Invasion analysis

We start with a population fixed for m and O, and ask whether G_1 (or, equivalently, G_2) can invade. First we calculate the equilibrium frequencies by solving $\hat{p}_i = F_i(\hat{\boldsymbol{p}}), i = 1\ldots4$:

$$\begin{aligned} \hat{p}_1 &= \hat{p}_4 = \tfrac{1}{2} - \tfrac{1}{2}d - \tfrac{1}{2}\varepsilon_O + d\varepsilon_O \\ \hat{p}_2 &= \hat{p}_3 = \tfrac{1}{2} - \hat{p}_1. \end{aligned} \quad \text{(A7)}$$

This equilibrium (the O-equilibrium) can be invaded by G_1 and G_2 if the dominant eigenvalue of the Jacobian matrix $(\partial F_i / \partial p_j)_{i,j=1..8}$ of (A6), restricted to $p_1 \ldots p_8$ and evaluated at (A7), is larger than 1. This is the case whenever

$$\varepsilon_O > \frac{s + 2d - 3sd - Z}{2s(1-2d)} = (1-s)\frac{d}{s} + O(d^2) \quad \text{(A8)}$$

where

$$Z = \sqrt{(s + 2d - sd)^2 - 4sd}. \quad \text{(A9)}$$

It turns out that there are no equilibria where the O-allele and G-alleles co-exist, hence O goes extinct after invasion of G-alleles, and a new equilibrium (the G-equilibrium) is reached:

$$\begin{aligned} \hat{p}_5 &= \hat{p}_8 = \frac{1}{4s}(s - 2d + sd + Z) \approx \tfrac{1}{2}(1 - d/s) \\ \hat{p}_6 &= \hat{p}_7 = \tfrac{1}{2} - \hat{p}_5 \end{aligned} \quad \text{(A10)}$$

In this equilibrium, the G-alleles are what we call *selection-based effects*, since they convey information about the environment of their carriers, build up by past selection. Indeed, it can be shown that in the G-equilibrium the correlation between G-alleles and the selective environment of their carriers is given by

$$r_{GE} = \frac{\text{cov}(G,E)}{\sqrt{V_G V_E}} = 4\hat{p}_5 - 1. \quad \text{(A11)}$$

The G–equilibrium can be invaded by O if the inequality in (A8) is reversed. It is very noteworthy that (A8) is in fact equivalent to

$$\hat{p}_5 > \hat{p}_1. \quad \text{(A12)}$$

In other words, the population evolves towards a state where the equilibrium frequency of well-matched phenotypes is maximized.

Now we ask if M can invade the G-equilibrium by inspecting the eigenvalues of $(\partial F_i / \partial p_j)_{i,j=5\ldots8,13\ldots16}$, and the result is that M invades whenever

$$\varepsilon_{\mathrm{M}} < (1-s)\frac{d}{s} + O(d^2) \qquad \text{(A13)}$$

Obviously, this is very similar to the reverse of condition (A8). When M has gone to fixation, the G-alleles correspond with what we call *detection-based transgenerational epigenetic effects*, since the G-alleles now convey selectively relevant information detected by the parent.

Reference

Berrigan, D. and Scheiner, S. M. 2004. Modeling the evolution of phenotypic plasticity. In T. J. DeWitt and S. M. Scheiner (eds), Phenotypic plasticity. Functional and conceptual approaches. Oxford University Press, New York, pp. 82-97,
